# Disparities in the Prevalence of Childhood Obesity-Related Comorbidities: A Systematic Review

**DOI:** 10.3389/fpubh.2022.923744

**Published:** 2022-07-06

**Authors:** George Obita, Ahmad Alkhatib

**Affiliations:** School of Health and Life Sciences, Teesside University, Middlesbrough, United Kingdom

**Keywords:** childhood obesity, comorbidity, disparity, prevalence, non-communicable disease

## Abstract

**Background:**

Non-communicable diseases among children are serious consequences of childhood obesity. However, less is known about the disparities in childhood obesity comorbidities burden. This review describes the salient pattern of disparities in the prevalence of childhood obesity-related non-communicable diseases and relevant inequalities in both high- and low/medium-income countries.

**Method:**

A systematic literature search was performed in MEDLINE, Embase, CINAHL, PsycInfo, Scopus, and Web of Science databases by two independent reviewers. Inclusion criteria were as follows: age 2–18 years; the prevalence or incidence of childhood obesity comorbidities reported; and studies published in English from January 2010 to date. No restrictions on the setting. The prevalence data were analyzed using range and median for subgroups based on the country's development status, gender, and geographical region.

**Results:**

Our search identified 6,837 articles, out of which we examined 145 full-text articles and included 54 articles in the analysis. The median prevalence of childhood obesity-related hypertension was 35.6 vs. 12.7% among middle- and low-income countries compared with high-income countries; 37.7 vs. 32.9% among boys compared with girls; and 38.6, 25.3, and 20.1% in Asia, South America, and Europe, respectively. For metabolic syndrome, the median prevalence was 26.9 vs. 5.5% among middle- and low-income countries compared with high-income countries; 55.2 vs. 12.0% among boys compared with girls; and 40.3, 25.8, and 7.7% in South America, Asia, and Europe, respectively. The prevalence of childhood obesity-related non-alcoholic fatty liver disease was 47.5 vs. 23% among middle- and low-income countries compared with high-income countries; and 52.1, 39.7, and 23.0% in Asia, South America, and Europe, respectively. The median prevalence of dyslipidemia was 43.5 vs. 63% among middle- and low-income countries compared with high-income countries; 55.2 vs. 12.0% among boys compared to girls; and 73.7 and 49.2% in Australia and Europe, respectively.

**Conclusion:**

There are disparities in the prevalence of childhood obesity-related hypertension, metabolic syndrome, and non-alcoholic fatty liver disease, with middle- and low-income countries, boys, and Asian region having higher prevalence. Implementing targeted interventions for childhood obesity comorbidities should consider socioeconomic disparities and strengthening of research surveillance methods for a better understanding of non-communicable disease burden in the pediatric population.

**Systematic Review Registration:**

https://www.crd.york.ac.uk/PROSPERO, identifier: CRD42021288607.

## Introduction

There is a growing burden of non-communicable diseases (NCD) among children and adolescents worldwide (1). Over 2.1 billion children and adolescents under the age of 20 years were estimated to be affected by NCD globally. These include cardiovascular diseases (CVD) (13.9 million), cancers (5.9 million), chronic respiratory disorders (108.9 million), type 2 diabetes mellitus (T2DM) (8.8 million), mental health disorders (231.3 million), and injuries and violence (170.4 million). Children with NCD face lifelong burden of illness and contribute to over one-thirds of adulthood NCD incidence (2). The rise in the risk of NCD in children has been associated with the increase in childhood obesity prevalence (3, 4). Childhood obesity rate increased over 3-fold worldwide over the past three decades (5), indicating a growing global epidemic of childhood obesity. It is estimated that about 10% of school-aged children worldwide contribute to childhood obesity with an increased risk of developing chronic NCD (2). Besides the increased risk of NCD, childhood obesity causes wider societal impact such as stigma and discrimination, increased care-related cost to the affected families, cost to the community through the diversion of resources for the treatment of obesity and related conditions, and the direct healthcare cost for treating obesity and related conditions. It is estimated that obesity constitutes l−5% of total healthcare costs in various countries (6).

The American Medical Association (AMA) and World Health Organization (WHO) recognized obesity as a distinct disease (7, 8). Moreover, the World Obesity Federation identifies it as a “chronic, relapsing, progressive, disease process” (9). Obesity as a disease entity may coexist with other conditions as part of multimorbidity (MM) or as an index condition in comorbidity (10–14). MM and comorbidity increase the complexities of long-term care of affected individuals (15). In this regard, the most commonly reported obesity comorbidities include hypertension, T2DM, non-alcoholic fatty liver disease (NAFLD), CVD, and a cluster of NCD such as metabolic syndrome.

The mechanism through which obesity leads to the development of NCD is complex, but it involves its inflammatory, oxidative stress, and insulin resistance effects (16). Insulin resistance leads to compensatory hyperinsulinemia, which in turn is responsible for most of the metabolic and cardiovascular comorbidities associated with obesity (17, 18). Insulin resistance is the first step in T2DM pathogenesis followed by impaired insulin secretion, which ultimately manifests as clinical T2DM (17–19). Insulin resistance is also part of the metabolic syndrome, characterized by hyperinsulinemia, insulin resistance, dyslipidemia, hypertension, and central obesity (20), and is implicated in gastrointestinal comorbidities such as NAFLD (21).

The association between obesity and NCD has been demonstrated in several observational studies (22–27). For example, Pantalone et al. (24) found that there was a higher prevalence of T2DM, pre-diabetes, hypertension, and CVD in higher body mass index (BMI) categories compared with lower BMI, *p* < 0.0001. A recent systematic review and meta-analysis of 52 studies demonstrated the association between childhood obesity and NCD, with prevalence ratios of 1.4 for hyperlipidemia, 21.2 for CVD, 26.1 for NAFLD, 1.7 for pulmonary disorders, and 4.0 for hypertension (23). The Bogalusa Heart Study, a long-term epidemiological study in Louisiana, USA, also showed that hypertension increased 8.5-fold and dyslipidemia increased from 3.1- to 8.3-fold in overweight adolescents as compared with healthy weight adolescents (28, 29). Therefore, obesity in childhood that persists in adolescence has a causal link with multiple NCD.

Disparities in childhood obesity prevalence are well documented, especially those related to ethnicity and socioeconomic status. For example, in a study of ethnic and race disparity in early childhood obesity, Zilanawala et al. (30) reported that, compared with white children, the odd of obesity was higher among black Caribbean children, odds ratio (OR) = 1.7 (95% CI: 1.1–2.6), whereas Pakistani children had a lower OR = 0.60 (95%CI: 0.37–0.96). On the contrary, black African children were more likely to be overweight, OR = 1.40 (95% CI: 1.04–1.88) (30). Falconer et al. (31) found that among school-aged children in England, a higher percentage of Asian and black children than white children were overweight or obese (21–27 vs. 16%), lived in the most deprived areas (24–47 vs. 14%), and reported lifestyle that leads to obesity (38 vs. 16%). With regard to socioeconomic factors, studies show that socioeconomic disadvantage is associated with childhood obesity that is sustained in subsequent generations (32). In a cohort of 22,810 participants, BMI was found to be higher in those in the lowest socioeconomic class than those in the highest socioeconomic class by 2.0 kg/m^2^ (*p* < 0.001) (33). Analysis of a nationally representative data in the United States (USA) showed that children from middle- and high-income households were 0.78 (95% CI: 0.72–0.83) and 0.68 (95% CI: 0.59–0.77) times more likely to be overweight or obese compared with those from low-income households (34). The United Kingdom's (UK) Millennium Cohort Study shows that at the age of 17 years, 27.6% of those from the lowest income quintile classify as obese compared with 13.7% (*p* = 0.001) of those from the richest households (35). These studies provide evidence of disparities in childhood obesity by ethnicity and socioeconomic status.

Despite ample evidence of disparities in childhood obesity, there is a lack of literature on disparities in the prevalence of childhood obesity-related comorbidities. Often, literature on childhood obesity-related comorbidities describes the association between overweight/obesity and comorbidities (23). In a few instances, gender disparity in prevalence is reported (36–38). Nevertheless, there are some indications of ethnic disparity in the prevalence of NCD among children with obesity. For example, Cheung et al. (39) showed that the prevalence of hypertension among obese adolescents differed by ethnicity, with a significant difference among Hispanic (3.1%), African Americans (2.7%), and white (2.6%) adolescents (*p* = 0.02). Although it is not clear whether and how disparities in childhood obesity-related comorbidities prevalence are based on socioeconomic status, recent systematic reviews have shown disparities in MM burden between low- and middle-income countries (LMICs) and high-income countries (HICs) (14, 40–42). Such analyses are essential for devising effective preventative approaches in community and primary care settings (41).

Because many kinds of NCD are associated with obesity, prevention and management of obesity and its related NCD have become the focus of attention worldwide (43). A good understanding of disparities in childhood obesity-related NCD prevalence among different population groups provides insight into potential reasons for the differences and helps to address the common risk factors. This supports the WHO global strategy of reducing NCD burden through an integrated prevention approach of risk factors at individual, family, community, and population level (44). However, currently, there is no systematic review that has analyzed disparities in childhood obesity-related comorbidities prevalence. While there is evidence of disparity in childhood obesity burden, such evidence cannot be extrapolated to explain the disparity in childhood obesity-related NCD prevalence. Therefore, this review aims to describe the salient pattern of disparities in the prevalence of childhood obesity-related NCD comorbidities between HICs and LMICs, by gender and geographical region, with a view to potentially inform the development of interventions that address the most at-risk groups worldwide.

## Method

### Search Strategy

The protocol for this review was registered with the International Prospective Register of Systematic Reviews (PROSPERO: CRD42021288607), and the Preferred Reporting Items for Systematic Reviews and Meta-Analyses (PRISMA) statement was followed (45). We conducted a systematic search in MEDLINE, Embase, CINAHL, PsycInfo, Scopus, and Web of Science using a combination of free text and medical subheadings (MeSH) terms. We also searched the general Internet using the Discovery portal and a list of reference of relevant articles. Initially, a specific search strategy for MEDLINE was developed (Search strategies are available *via* the PROSPERO registry). This was then adapted to other databases. The search terms used were as follows: (children or adolescents or pediatric or students or school pupils or youth or boys or girls or school age or juvenile or preteens or teens) AND (obesity or body weight or adiposity or body mass index or waist circumference or neck circumference) AND (comorbidity or Type 2 Diabetes Mellitus or hypertension or high blood pressure or cardiovascular disease or CVD or metabolic syndrome or non-alcoholic fatty liver disease or NAFLD or depression or psychological problem or anxiety or self-esteem or sleep apnea or asthma or respiratory problem or dyslipidemia or musculoskeletal problems) AND (prevalence or incidence or odds ratio or risk ratio or occurrence or epidemiology).

### Inclusion and Exclusion Criteria

The inclusion criteria for selecting studies were as follows: (1) Participants were children or adolescents aged 2–18 years, based on the WHO definition of childhood obesity (3); however, some countries used above 18 years as childhood cutoff point (46). (2) Data that were reported on the prevalence or incidence of comorbidities or relevant information could be used to generate reliable prevalence estimates of childhood obesity-related NCD comorbidities. (3) The studies used a cross-sectional or case–control or prospective cohort design or randomized control trial where prevalence or incidence data were reported. (4) Only studies published in English or translated into English from January 2010 were included to reflect more current information. (5) There were no restrictions on the setting. Studies reporting prevalence/incidence of childhood obesity comorbidity at national or specific setting such as the community, school setting, or primary care worldwide were included.

Studies were excluded if they (1) were conducted in selected population groups, such as those identified through special clinics and children with obesity as a symptom of an underlying condition, for example, Prader-Willi syndrome, Cushing syndrome, hypothyroidism, and Hashimoto disease, or a side effect from medication such as antipsychotics; (2) were case series, opinion papers, and all types of qualitative studies. Whenever a particular dataset was published more than once, the most recent publication was used.

### Study Selection, Quality Assessment, and Data Extraction

Two reviewers screened the title and abstract of retrieved articles to exclude studies that were not eligible. The two reviewers independently appraised the full text of studies that met the inclusion criteria. Any discrepancies were resolved through consensus. Search results are reported according to the Preferred Reporting Items for Systematic Reviews and Meta-analyses (PRISMA) flow diagram (47), including reasons for excluding a full-text study. The reviewers were not blind to the journal titles.

The two reviewers assessed the quality of selected studies independently using the Joanna Briggs Institute's (JBI) Critical Appraisal Checklist for Studies Reporting Prevalence Data (48, 49), adopting a scoring system of 0–5 for poor quality and 6–10 for high quality. The checklist assesses sample representativeness, reliability of measurement, and whether sufficient details of weight classification and comorbidity definition were reported. Each of the two reviewers independently assessed the quality of each selected article. Discrepancies were resolved through consensus. Poor-quality studies were excluded from the analysis.

A single reviewer extracted data regarding the first author, year of publication, study design, country, sample size, participants' age group, type of comorbidity, diagnosis criteria, and the reported prevalence or incidence into a spread sheet. The second reviewer examined the extracted data and any discrepancies were resolved through discussions.

### Data Synthesis and Analysis

The extracted data were first grouped by the countries' developmental/income status (HICs vs. LMICs, according to the United Nations development status, 1999 and World Bank classification of countries by income) (50, 51); geographical region (North America, South America, Europe, Asia, Africa, and Australasia); and gender (male vs. female). Comorbidities were grouped into the following categories: (1) Metabolic syndrome, (2) dyslipidemia, (3) hypertension, (4) NAFLD, (5) pulmonary disorder, (6) psychological comorbidities, and (7) other comorbidities. To compare the prevalence of a specific comorbidity between subgroups, descriptive statistics of range and median were used to summarize the prevalence estimate of the subgroups within development/income status, geographical region, and gender (52). SPSS version 26 was used to calculate the median based on the formula: [(n + 1) ÷ 2]th, where “n” is the number of items in the set and “th” just means the (n)th number (52). Meta-analysis was not possible because the studies were not sufficiently homogeneous in terms of methodology, participants' age groups, and measurement.

## Results

### Search Results

**Figure 1:**
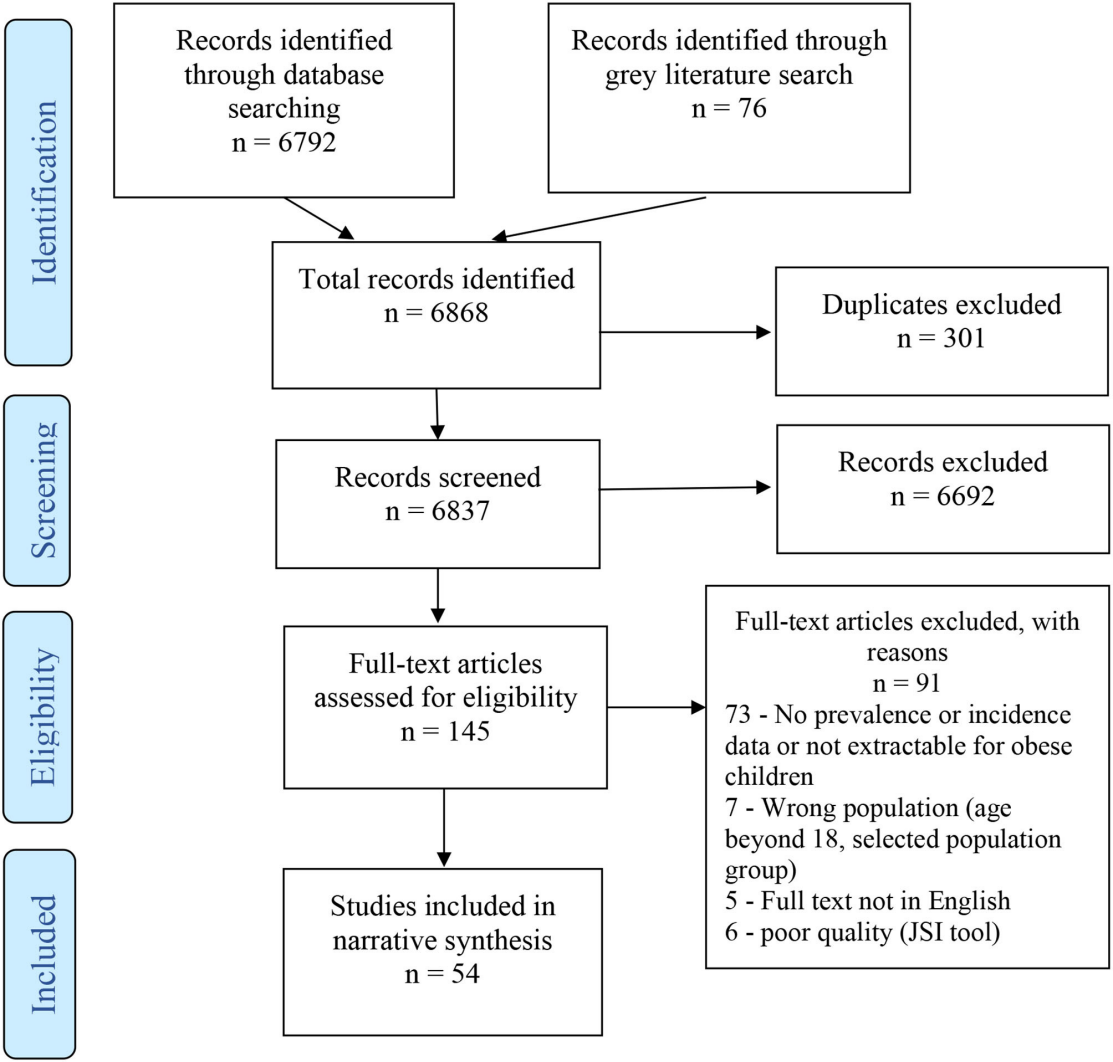
Flowchart of study selection based on Preferred Reporting Items for Systematic Reviews and Meta-Analysis (PRISMA) guidelines.

The search identified a total of 6,868 articles, 301 of which were duplicates. Titles and abstracts of 6,837 articles were screened, resulting in 145 full texts being examined for eligibility. Totally, 54 of the examined full texts met our inclusion criteria (Figure 1).

### Characteristics of Selected Studies

The 54 selected studies that described the prevalence of childhood obesity comorbidities in the obese weight categories included a total of 651,659 participants. The 54 studies were conducted in 27 countries across five continents, with 27 articles from Asia, 17 from Europe, six from South America, two from North America, one from Africa, and one from Australasia (Table 1). The most commonly reported comorbidity was hypertension, followed by metabolic syndrome (NB includes hypertension) and dyslipidemia. The least reported comorbidities were asthma and emotional disorders (Table 1).

**Table 1:** Childhood obesity comorbidities prevalence by geographical region.

**Childhood obesity comorbidity**	**Continental region**	**Prevalence range**	**Prevalence median**
Hypertension	Asia	12.0–61.7%	38.6%
	South America	18.8–30.9%	25.3%
	Europe	8.6–48.8%	20.1%
	North America	8.2–8.2%	8.2%
	Africa	No studies	No studies
	Australasia	No studies	No studies
Metabolic syndrome	South America	26.3–72.8%	40.3%
	Asia	6.6–52.1%	25.8%
	Europe	7.7–33.0%	20.4%
	Africa	14.3–14.3% (single study)	14.3%
	Australasia	No studies	No studies
	North America	No studies	No studies
NAFLD	Asia	45.0–52.1%	48.6%
	Europe	9.5–36.4%	23.0%
	South America	26.0–50.0%	39.7%
	Africa	No studies	No studies
	Australasia	No studies	No studies
	North America	No studies	No studies
Dyslipidemia	Europe	43.5–54.0%	48.8%
	South America	52.0–74.0%	63.0%
	North America	No studies	No studies
	Africa	No studies	No studies
	Australasia	No studies	No studies
	Asia	No studies	No studies
Asthma	North America	11.6–11.6% (single study)	11.6%
	Europe	No studies	No studies
	South America	No studies	No studies
	Africa	No studies	No studies
	Australasia	No studies	No studies
	Asia	No studies	No studies
Anxiety-depression	Asia	30.9–30.9% (single study)	30.9%
	Europe	16.8–16.8% (single study)	16.8%
	North America	No studies	No studies
	South America	No studies	No studies
	Africa	No studies	No studies
	Australasia	No studies	No studies
	Antarctica	No studies	No studies

*NAFLD, Non-alcoholic fatty liver disease*.

While a few studies provided separate estimates for children (≤ 10 years) and adolescents (≥10 years) (53, 54), the majority reported combined prevalence, with ages ranging from 2 to 18 years. This means that the prevalence of different age subgroups could not be compared. Eleven papers reported assessing prevalence by gender, but only two provided breakdowns by gender and age group. All the studies were observational in design with the majority being cross-sectional surveys. Table 2 summarizes the characteristics of included studies.

**Table 2:** Characteristics of studies that described childhood obesity comorbidities.

**Reference**	**Study design**	**Sample size (number)**	**Country**	**Age group (years)**	**Comorbidity type**	**Diagnosis criteria**	**Prevalence in children with obesity**	**Quality score**
China Medical Association (55)	Cluster sample survey	22,071	China	7–16	Metabolic syndrome	International Diabetes Federation (IDF) Criteria	16.8%	6
Badeli et al. (56)	Cross-sectional survey	2,072	Iran	7–17	Hypertension	SBP and/or DBP 95th percentile for age, sex, and height	51.5% (M) 12.5% (F)	8
Basiratnia. et al. (57)	Cross-sectional survey	2,000	Iran	11–17	Hypertension	SBP and/or DBPs > 95th percentile for age, sex, and height	30.7%	7
Cheng. et al. (58)	Prospective cohort	2,189	China	6–16	Hypertension	SBP and/or DBP > 95th percentile for age-sex-height	32.4%	6
Cheng et al. (59)	Multi-stage Cluster-sample survey	1,309	China	10–17	Metabolic syndrome	According to Metabolic syndrome and prophylaxis and treatment proposal in Chinese children and adolescents	41.2%	6
Dong et al. (60)	Cross-sectional survey	4,898	China	6–17	Hypertension	The age- and gender-specific BP cutoff points in Chinese children and adolescents	38.7%	9
Esposito et al. (61)	Case-control study	148	Italy	79	Anxiety-depression	CDI	16.8%	6
Genovesi et al. (62)	Cross-section survey	5,131	Italy	5–11	Hypertension	When SBP and/or DBP at first screening were > or = 90th percentile and the mean of three subsequent measures was > or =95th percentile.	21.5% (M) 20.1% (F)	6
Jmal et al. (63)	Cross-sectional survey	306	Tunisia	10–12	Metabolic syndrome	IDF criteria.	14.3%	7
Koebnick et al. (64)	Cross-sectional survey	237,238	USA	6–17	Hypertension	Two BP ≥ 95th percentile (or ≥140/90 mm Hg even if lower than the 95th percentile	8.2%	8
Luo et al. (65)	Cross-sectional survey.	7,893	China	6–18	Hypertension	SBP and/or DBP > 95^th^ Percentile	19.2%	8
Manios et al. (66)	Cross-sectional survey	2,263	Greece	13–18	Hypertension	SBP and/or DBP > 95^th^ Percentile	48.3%	8
Nkeh-Chungag et al. (67)	Cross-sectional survey	388	Czech Republic	13–17	Hypertension	SBP and DBP ≥ 95th percentile for height, age and sex	17.7%	7
Ogunleye et al., [2013]	Cross-sectional survey	5,983	England	10–16	Mean arterial pressure (MAP)	MAP = 2/3 DBP + 1/3 SBP	29.7%	6
Pećin et al. (68)	Cross-sectional survey	750	Croatia	15.9	Hypertension	According to the current ESH/ESC guidelines	20.0%	9
Pontiles de Sánchez. et al. (69)	Cross-sectional survey	85	Venezuela	3–6	NAFLD	Fatty liver pancreas-US	50.0%	8
				7–11	NAFLD	Fatty liver pancreas-US	39.7%	
				12–17	NAFLD	Fatty liver pancreas-US	31.6%	
Rakočević et al. (70)	Cross-sectional	173	Croatia	7–16	Hypertension	BP = > 130/85 mm Hg	25%	6
Sangun et al. (71)	Records review	614	Turkey	7-18	Metabolic syndrome	IDF criteria	33.0%	7
Saury-Paredes et al. (72)	Cross-sectional.	259	Mexico	5–11	Hypertension	SBP and/or DBP ≥ 95^th^ percentile for gender, age	18.8%	8
Shirasawa et al. (73)	Cross-sectional survey	1,297	Japan	9–10	Hypertension	an SBP ≥ 135 mm Hg or DBP ≥ 80 mm Hg	39.4% (M) 58.1% (F)	9
		1,088		12–13	Hypertension	an SBP ≥140 mm Hg or DBP ≥ 85 mm Hg was defined as HT in boys, while an SBP ≥ 135 mm Hg or DBP ≥ 80 mm Hg was defined as HT in girls.	35.7% (M) 38.5% (F)	
Steinthorsdottir et al. (74)	Cross-sectional survey	1,071	Iceland	9–10	Hypertension	BP ≥ 95th percentile at all three visits	8.6%	8
Suarez-Ortegón et al. (75)	Cross-sectional	1,461	Colombia	10–16	Metabolic syndrome	IDF criteria	72.8%	7
Suazo et al. (76)	Cross-sectional survey	259	Chile	6–12	Metabolic syndrome	the definitions by Cook et al.	26.3%	7
Xu et al. (54)	Cross-sectional survey	8,764	China	7–11	Metabolic syndrome	IDF criteria	33.7% (>10 yrs) 21.8% (<10 yrs)	6
Zhang et al. (77)	Cross-sectional survey	38,702	China	7–17	Hypertension	SBP and/or DBP ≥ 95th percentile for age and gender	60.7 (M) 58.2% (F)	8
Zhang et al. (78)	Cross-sectional survey.	8,568	China	7–18	Hypertension	The 95th percentile of BP cutoff	57.8%	7
Zhang et al. (79)	Cross-sectional survey.	38,822	China	7–17	Hypertension	SBP and/or DBP C 95th percentile for age and gender	48.6% (M) 38.8% (F)	6
Zhou et al. (37)	Cross-sectional survey	387	China	12–17	NAFLD	Diagnostic criteria recommended by the Fatty liver and Alcoholic Liver Disease Study Group of Liver Disease Association in China	45.0%	8
Rerksuppaphol et al. (80)	Cross-sectional survey.	3,991	Thailand	4–17	Hypertension	SBP and/or DBP ≥95th percentile for age and gender	49.5	6
Önsüz et al. (53)	Cross-sectional survey	2,166	Turkey	6–15	Hypertension	Having the average SBP) or DBP between the 95th percentile and the 99th percentile for sex, age, and height	27.7% (All) 23.4% (M <10) 43.5% (M>10) 21.9% (F <10) 36.0% (F>10)	9
Minghelli et al. (81)	Cross-sectional survey	966	Portugal	10–16	Hypertension	SBP or DBP > 95th percentile	12.7%	9
					Lower back pain		6.1%	
Elmaogullari et al. (82)	Cross-sectional (Retrospective record review)	823	Turkey	2–18	Dyslipidemia	Dyslipidemia criteria	42.9%	9
					NAFLD	USS	56.4%	
Sukhonthachit et al. (83)	Cross-sectional survey		Thailand	8–12	hypertension	SBP and/or DBP ≥ 95th percentile for hypertension	13.8%	9
Lim et al. (84)	Cross-sectional survey	1,526	Korea	10–19	Hypertension	SBP and/or DBP ≥ 95th percentile for hypertension	28.4%	9
					Metabolic syndrome	2007 IDF criteria	24.7%	
					Dyslipidemia		55.4%	
					High glucose		9.2%	
Elizondo-Montemayor et al. (85)	Cross-sectional survey	236	Mexico	6–12	Metabolic syndrome	Cook's criteria	40.3%	6
Dyson et al. (86)	Cross-sectional survey.	12,730	China	12–18	Hypertension	Mean SBP or DBP reading (or both) 95th per centile for the predicted value based on gender, age and height	21.0% (M) 29.7% (F)	9
			India	12–18	Hypertension		44.4% (M) 50.0% (F)	
			Mexico	12–18	Hypertension		30.9% (M) 25.3% (F)	
Wang et al. (87)	Cross-sectional survey	3,373	China	16–18	Hypertension		25.5% (M) 11.0% (F)	6
					Metabolic syndrome	IDF definition >=10yrs	14.7% (M) 13.7% (F)	
						Cook's <10yrs	18.8% (M) 26.9% (F)	
						Cook's >=10yrs	33.7% (M) 30.5% (F)	
Mehairi et al. (88)	Cross-sectional survey	1,018	UAE	12–18	Metabolic syndrome	IDF criteria	55.2% (M) 12.0% (F)	6
Gong et al. (89)	Cross-sectional survey.	538	China	9–15	NAFLD	USS	52.1%	8
Zakeri et al. (90)	Cross-sectional survey	9,172	Iran	10–18	Emotional problems	Global School-based Health Survey (GSHS)	24.0%	6
					Depressive problems	Global School-based Health Survey (GSHS)	30.9%	
					Anxiety problems	Global School-based Health Survey (GSHS)	9.7%	
Papoutsakis et al. (91)	Prospective study	1,138	Greece	10–14	Metabolic Syndrome	IDF	7.7%	6
					Elevated BP		55.0%	
Chen et al. (92)	Cross-sectional survey	3,814	China	6–18	Metabolic syndrome	IDF criteria	27.6%	6
Wiegand et al. (93)	Cross-sectional survey	16,390	The Netherland	1–20	NAFLD	elevated AST and/or ALT	9.5%	5
					Hypertension	95th percentile of European reference data	16.7%	
					Hyperlipidemia	according to the American Heart Association	36.8%	
Rafraf et al. (94)	Cross-sectional survey	985	Iran	14–17	Hypertension	SBP and/or DBP ≥ 95th percentile	46.4%	6
Noonan et al. (95)	Cross-sectional survey	1,852	USA	9–18 (4^th^-12^th^ graders)	Asthma	Questionnaire: (physician or other health professional had told them they had asthma)	11.6%	6
Kloppenborg et al. (96)	Cross-section survey	3,978	Denmark	9–15	IFG	WHO definition	3.4 (F) 4.1 (M)	6
Di Bonito et al. (97)	Multi-center cross-section records review	1,769	Italy	5–18	NAFLD	USS	36.4%	6
Sadeghi-Demneh et al. (98)	Multi-level cluster survey	667	Iran	7–14	Flexible foot	Clinical assessment	52.8%	9
	Multi-level cluster survey				Rigid foot	Clinical assessment	25.0%	6
	Multi-level cluster survey				Activity pain	Clinical assessment	3.2%	6
Schwandt et al. (99)	Analysis of secondary data	22,051	Germany	3–18	Hypertension		18.6 (F) 24.0% (M)	6
Rerksuppaphol et al. (80)	Cross-sectional survey	3,991	Thailand	4–17	Hypertension		49.5	6
Kajbaf et al. (100)	Cross-sectional survey	903	Iran	7–11	Wheeze in the past		68.7%	9
Kajbaf et al. (100)	Cross-sectional survey				Exercise induced wheeze		23.4%	6
Bell et al. (101)	Case control- incidence study	283	Australia	6–13	IGT		5.3%	6
					Hyperinsulism		38.9%	
					Hypertension		19.0%	
					Dyslipidemia		73.7%	

*ALT, Alanine transferase; AST, aspartate transferase; BP, blood pressure; CDI, The Children Depression Inventory (CDI); DBP, diastolic blood pressure; F, female; GSHS, Global School-based Health Survey; IDF, International Diabetes Federation; IGT, impaired glucose test; M, male; MAP, mean arterial pressure; NAFLD, non-alcoholic fatty liver disease; SBP, systolic blood pressure; USS, ultra sound scan; WHO, World Health Organization*.

#### Disparity in Childhood Obesity Comorbidity Based on Geographical Ethnicity and Country

There was evidence of disparities in childhood obesity-related comorbidity prevalence between HICs and LMICs as shown in Table 3. The prevalence of hypertension in LMICs ranged from 13.8 to 60.7%, with a median of 35.6%, whereas in the HICs it ranged from 3 to 26% with a median of 12.7%. The highest hypertension prevalence, 60.7%, was reported among Chinese males (109), followed by Indian girls at 50% (86), then Thai children at 49.5 % (80), and Turkish males at 43.5% (110).

**Table 3:** Childhood obesity comorbidity prevalence range in children aged 2–18 years by development status.

**Childhood obesity comorbidity**	**Range of prevalence of obesity comorbidity in HICs (2 to 18 years)**.	**Range of prevalence range of obesity comorbidity in LMICs (2 to 18 years)**.	**Median of prevalence of obesity comorbidity in HICs (2 to 18 years). (95% CI)**	**Median of prevalence of obesity comorbidity in LMICs (2 to 18 years) (95% CI)**	**Comments**
Hypertension (SBP and/or DBP > 95th percentile)	3–26% (62, 64, 74, 81, 84, 93, 99, 102–104)	14–61% (36, 53, 56–58, 60, 65–68, 70, 72, 77, 78, 80, 83, 86, 87, 105–107)	13%	36%^*^	Higher prevalence of hypertension among children in Thailand, Turkey, China and Mexico than in any of the Western developed countries
MetS (IDF criteria)	3–8% (96, 102)	12–73% (55, 59, 63, 71, 75, 76, 84, 87, 88, 92)	6%	27%^**^	Children from Colombia aged 10–16 had highest prevalence (~73%) compared to highest the developed countries, Greece at 8%
NAFLD (steatosis echogenicity)	10–36% (93, 97)	32–56% (37, 69, 82, 89)	23%	48%^**^	Higher prevalence of NAFLD in developing countries
Dyslipidemia (fasting lipids > 95th percentile)	52–74% (84, 101)	43–54% (82, 108)	63%	44%^**^	Highest prevalence of dyslipidemia was in Australian children
Anxiety–Depression (CDI/ GSHS)	10–17% (61, 103)	10% (90)	13%	10%	No discernable trends

*BP, Blood pressure; CDI, The Children Depression Inventory; CI, confidence interval; DBP, diastolic blood pressure; GSHS, Global School-based Health Survey; IDF, International Diabetes Federation; NAFLD, non-alcoholic fatty liver disease; SBP, systolic blood pressure*.

* *p < 0.05;*

** *p > 0.05*.

Similarly, the prevalence of metabolic syndrome among children with obesity was shown to be higher among the LMICs, with a range of 12 to 72.8% and a median of 26.9%, compared with a range of 3 to 8% and a median of 5.5% in HICs. Colombian children had the highest prevalence of metabolic syndrome at 72.8% (108), followed by Chinese children with a prevalence of 41% and Mexican children at 40% (59, 85). Among HICs, Italian children had the highest prevalence of metabolic syndrome at 8.2% and Danish children had the lowest prevalence at 3.1% (96, 102).

The prevalence of childhood obesity-related NAFLD was higher among children from LMICs with a prevalence range of 31.6 to 56.4% and a median of 47.5%, compared with a range of 9.5–35.4% and a median of 23% in HICs. Up to 56.4% of Turkish children were reported to have NAFLD (82). The highest prevalence among HICs, 35.4%, was reported among Italian children (97).

The prevalence pattern of dyslipidemia, however, differed from the other comorbidities. Higher prevalence of dyslipidemia was reported among the HICs with a range of 52 to 74% and a median of 63% compared with a range of 43 to 52% and a median of 43.5% in LMICs. Canadian and Australian children had a prevalence of 52 and 74%, respectively (101, 103), while Turkish and Chilean children had a prevalence of 43 and 54%, respectively (82, 108).

A limited number of studies reported respiratory problems (asthma and wheezes) among children with obesity (95, 100), as well as the prevalence of psychological problems of anxiety and depression (90). However, disparity was difficult to discern for these comorbidities due to the small number of studies that met the inclusion criteria.

#### Disparity of Childhood Obesity Comorbidities by Gender

In total, eleven of the included studies disaggregated data by gender for the comorbidities of hypertension and metabolic syndrome. Nine reported on hypertension disaggregated by gender (53, 56, 62, 73, 79, 86, 87, 99, 111). Hypertension prevalence among girls ranged from 11.0 to 58.2% with a median of 32.9%. Among boys, the range was 21.0 to 60.7% with a median of 37.6% (Table 4). Only two studies reported metabolic syndrome by gender (87, 88). One of the studies showed a marked difference in the prevalence of metabolic syndrome between boys and girls (55.2 vs. 12.0%) (88).

**Table 4:** Childhood obesity comorbidity by gender.

**Obesity comorbidity**	**Prevalence**	**Prevalence**	**Prevalence**	**Prevalence**
	**range boys**	**range girls**	**median boys**	**median girls**
Hypertension	21.0–60.7%	11.0–58.2%	37.6%	32.9%
Metabolic syndrome	14.7–55.2%	12.0–13.7%	35.0%	12.9%

#### Disparity of Childhood Obesity Comorbidities by Global Regions

The range of the prevalence of hypertension among obese children in Asia was 11.0 to 60.7%, median 38.6%, compared with 18.8 to 30.9%, median 25.3% in South America, and 8.6 to 55.0%, median of 20.1% in Europe. North America had only one study that report on hypertension, whereas Africa, Australia, and Antarctica had none.

The prevalence range of metabolic syndrome was 26.3 to 72.8%, median 40.3% in South America; 6.6 to 52.1%, median 25.8% in Asia; and 7.7 to 33.0%, median 7.7% in Europe. One study reported on metabolic syndrome from North Africa with a prevalence of 14.3% (63), while none was reported from the remaining global regions (Australasia and North America).

With regard to NAFLD, the prevalence ranged from 45.0 to 52.1%, median 52.1% in Asia; 26.0 to 50.0%, median 39.7% in South America; and 9.5 to 36.4%, median 23% in Europe. The other regions had no studies reporting on NAFLD. For anxiety/depression, only one study was reported from Asia, with a prevalence of 30.9%, and one from Europe, with a prevalence of 16.8%. Similarly, two studies reported dyslipidemia from Europe, prevalence 43.5 to 54%, median 48.8%, and two from South America, prevalence 52.0 to 74%, median 63%. One study in North America reported on Asthma, prevalence 11.6% (95).

## Discussion

The main findings of this review were that the prevalence of childhood obesity-related hypertension, metabolic syndrome, and NAFLD were higher among populations in LMICs than those in HICs. Gender disparity was also apparent among children with obesity-related hypertension and dyslipidemia, where the prevalence was higher among boys than girls. Globally, the Asian region had the highest prevalence of childhood obesity-related hypertension followed by South America and then Europe. The prevalence of childhood obesity-related metabolic syndrome was highest in South America, followed by Asia and then Europe.

Explaining the observed disparities in childhood obesity-related comorbidities is expected to be as complex as defining populations' obesity disparity because of the intricate interplay between adiposity and known NCD risk factors, such as biological, environmental, lifestyle, genetic, socioeconomic, racial, and cultural factors. Nevertheless, understanding the prevalence of obesity-related NCD across pediatric populations, through the review of studies that described childhood obesity comorbidities (Table 2), is likely to provide evidence that could help develop strategies to ameliorate obesity and associated health risks in the pediatric population.

The review showed that LMICs and global regions with lower income status had higher prevalence of the common childhood obesity-related NCD than HICs (Tables 1, 3). This is consistent with reports that populations living in LMICs or having low socioeconomic status in HICs are at increased risk of developing NCD such as CVD, NAFLD, and T2DM (112–114). For example, the adjusted incidence of stroke was reported to have doubled from 52 to 117 per 100,000 person-years in LMICs, but decreased in HICs by about 42% over a 4-year period (113). Furthermore, Sposato et al. (114) also found that lower per capita gross domestic product adjusted for purchasing power parity correlated with a higher incident risk of stroke (*p* = 0.027, *R*^2^ = 0.32) (114). While it is estimated that the global NCD burden will increase by 17% in the next 10 years, in the African region it is projected to increase by up to 27% (5). Similarly, almost half of all deaths of all ages in Asia are now attributable to NCD, accounting for 47% of the global burden of disease (5). At present, India is projected to have the highest global number of patients with diabetes, at 79.4 million in 2030 (115). Therefore, understanding NCD burdens specific to LMICs in the pediatric populations could help in devising effective prevention strategies for childhood obesity and its consequences based on the understanding of challenges found in LMICs. Such prevention challenges that may be shared with high-risk adult population include poverty, increased urbanization, lifestyle factors, and poor air quality, which are common in LMICs (14).

Although evidence suggests that living in LMICs is associated with an increased risk of developing NCD, the causal pathway between socioeconomic status and NCD is complex and not well understood (116). Some studies suggest that earlier adoption of healthy behaviors by advantaged socioeconomic groups is followed by an increased prevalence of risky behaviors among the disadvantaged socioeconomic groups (117, 118). For example, Marins et al. (119) reported a strong association between a low level of schooling and cardiovascular risk factors in an urban center in Brazil (OR1·77, 95% CI 1·39–2·26). While there is no conclusive explanation of the causal pathway between socioeconomic status and NCD, our analysis shows that children from LMICs are at more risk of NCD (Table 4). The reasons for this observed pattern may include material deprivation, exposure to unhealthy living conditions and unsafe environment, and limited access to high-quality health services and prevention interventions (120, 121). Adopting NCD prevention through awareness and behavioral interventions in LMICs should therefore adopt an economic-based model, in which community disparities are addressed. Such an approach was recently suggested for preventing MM in LMICs through targeting a cluster of NCD with a multipronged intervention approach (14). This may be adopted for specific age groups, including children with obesity, especially personalized interventions that prevent several metabolic risk factors (122, 123).

Several studies have reported that rapid urbanization and air pollution are linked to NCD in LMICs for all adults (124–130), and hence, children might be particularly disadvantaged in this regard and likely to benefit from early interventions that are appropriate for LMICs. In addition, relative poverty and its associated adverse effects on intrauterine environment affect the functional development of a fetus and lead to an increased risk of development of NCD in adolescence and later life (131). Epidemiological studies have linked small size births with increased risk of CVD and other NCD (132). Insufficient nutrition in the intrauterine life may result in increased susceptibility to lifestyle-related NCD risk factors (133). This is an important factor in childhood NCD burden in many LMICs, where the realities are increased urbanization associated with high levels of urban poverty and marginalization of the rural poor (134, 135). These might, in part, explain the observed differences in childhood obesity-related hypertension, metabolic syndrome, and NAFLD prevalence between LMICs and HICs reported in this review (Table 3).

In terms of ethnic and cultural disparity, ethnic minority groups within HICs are known to have a higher risk of obesity and associated NCD such as insulin resistance and metabolic disease (118). Several studies have reported the interplay among lifestyle, environmental, and genetic factors to explain a higher degree of hyperinsulinemia and correlation with adiposity, among South Asian compared with white children of similar age (131, 136–138). However, explaining disparities due to geographical ethnicity or country found in this review (Table 2) is complex. Biologically mediated racial or ethnic differences in NCD risks are reported but the actual genetic differences remain unclear as putative genes or gene variants have not yet been identified (139). However, exposure to NCD during fetal life and infancy is reported to increase the risk of developing childhood NCD (140). The Bogalusa Heart Study, for example, shows that the offspring of diabetic parents displays quicker progression to insulin resistance characteristics in the early years to adolescence (25), providing plausible evidence of biological factors at play. High prevalence of adult NCD among some population groups, therefore, increases the risk of childhood NCD in their offspring.

Interestingly, the trend in the prevalence of dyslipidemia departed from other childhood obesity-related NCD between HICs and LMICs in this review (Table 3). Although some previous studies in adults have shown lower levels of lipids in ethnic minority groups than in white counterparts, for instance, the mean plasma triglyceride concentration was estimated to be 20 mg/dl lower in African-Americans compared with their white counterparts (141), several studies have also shown that dyslipidemia and excessive body fat occur at lower levels of BMI in South Asians than in white ethnic group (131, 142–144). Given that there were only four studies that reported on dyslipidemia in this review, the differences may not reflect the true picture of disparity.

Our results on gender disparity in childhood comorbidity (hypertension and metabolic syndrome) in boys than girls are irrespective of whether they occurred within LMICs or HICs. This is consistent with previous reports on gender disparity in obesity among children. World Obesity Federation Atlas of Childhood Obesity (2019) showed that about 65% of countries reported a higher prevalence of obesity among boys than girls aged 5–19 years, most of which were in high- and middle-income countries (3). Furthermore, the national Canadian data 2004–2013 showed a 2-fold higher prevalence of obesity among boys than girls (145). Similarly, in China, among children aged 7–18 years, boys had a higher prevalence of obesity than girls (146). In the UK, the National Child Measurement Program (NCMP) shows that boys, children from most deprived areas, and ethnic minorities have a disproportionately higher prevalence of obesity (147). These differences are attributed to risk factors shared with other NCD such as biological differences in body composition and sociocultural differences between sexes. For example, girls in the HICs are reported to prefer food lower in energy, while boys consume more meat and energy-dense food (148). The findings of the review are therefore consistent with the expected gender disparities in obesity-related NCD.

### Implications for Practice and Research

Given the disparity in burden and underlying risks factors of childhood obesity comorbidities, the key implications for practice and research are summarized in Box 1.

Box 1 **Implications for practice and research.**

- The need for interventions that target at-risk population groups to be culturally and contextually sound through the involvement of specific population group. This should take into consideration the socioeconomic realities of the specific population, informed by local evidence and cultural appropriateness.
- The need to design and conduct research studies on how best to reach the at-risk population with an individualized approach in countries with resource constraints and weak health systems.
- The need to strengthen methods for surveillance of childhood obesity-related NCD to guide local policies and interventions. The WHO STEP-wise survey approach has a limitation when it comes to children as it targets adults aged 25–64 years (149). Development of tailored methodological approaches that take into consideration childhood obesity-related NCD is therefore required.

### Limitations

First of all, we included children aged 2 and 18 years based on the WHO definition of childhood obesity (3). However, some countries adopt a higher cutoff age for the definition of childhood obesity (46). As a result, we had to exclude some articles because they did not meet our inclusion criteria for the cutoff age. Second, each of the 54 selected studies used different methodological approaches in selecting study population, study setting, and survey methods (national survey, local community schools). Thus, it was not possible to perform a meta-analysis; instead, we provided median prevalence for predefined subgroups. Nevertheless, it was possible to draw several useful trends from the complex datasets of heterogeneous studies. Third, the majority of studies on LMICs seemed to have assessed hypertension more frequently than other NCDs, perhaps due to resource limitations. Consequently, this may have biased the true picture of the distribution of childhood obesity-related NCD in LMICs. Research using advanced screening in LMICs is therefore needed to provide a comprehensive picture of childhood obesity comorbidities burden.

## Conclusion

Childhood obesity, with its associated NCD comorbidities, is a major global public health problem. Globally, there is a disparity in the prevalence of childhood obesity-related hypertension, metabolic syndrome, dyslipidemia, and NAFLD comorbidities, between HICs and LMICs, different global regions, and genders. Socioeconomic factors seem to be the main determinant for disparity between LMICs and HICs, besides biological, environmental, cultural, and modifiable lifestyle differences. Implementing targeted lifestyle interventions that are context specific to the socioeconomic realities of the population and informed by local evidence is required. Furthermore, strengthening research and surveillance methods for childhood obesity-related NCD to improve local policies and appropriate interventions is needed.

## Data Availability Statement

The original contributions presented in the study are included in the article/Supplementary Material, further inquiries can be directed to the corresponding author.

## Author Contributions

GO and AA contributed to conception, design of the study, performed article screening, and quality assessment. GO organized literature search and wrote the first draft of the manuscript. AA critically revised and edited the manuscript. Both authors reviewed and approved the finally submitted version.

## Conflict of Interest

The authors declare that the research was conducted in the absence of any commercial or financial relationships that could be construed as a potential conflict of interest.

## Publisher's Note

All claims expressed in this article are solely those of the authors and do not necessarily represent those of their affiliated organizations, or those of the publisher, the editors and the reviewers. Any product that may be evaluated in this article, or claim that may be made by its manufacturer, is not guaranteed or endorsed by the publisher.
