# Supplementary material for: Disparities in the Prevalence of Childhood Obesity-Related Comorbidities: A Systematic Review
Source: Front Public Health. 2022 Jul 6;10:923744. doi: 10.3389/fpubh.2022.923744 (PMC9298527; doi:10.3389/fpubh.2022.923744)
Supplement: Supplementary file 1 [file Data_Sheet_1.pdf]

## Search strategy and results from the databases

Supplementary Tables 1-6 showing results from each database: MEDLINE (Table 1), EMBASE (Table 2), CINAHL (Table 3), PsycInfo (Table 4), Scopus (Table 5) and, Web of Science Table 6).

| Table 1: Searches in Medline database | | |
|---|---|---|
| **#** | **Query** | **Results** |
| S71 | S19 AND S49 AND S69 AND S70 | 3,847 |
| S70 | S50 OR S51 OR S52 OR S53 OR S54 OR S55 OR S56 OR S57 OR S58 | 6,014,642 |
| S69 | S50 OR S51 OR S52 OR S53 OR S54 OR S55 OR S56 OR S57 OR S58 OR S59 OR S60 OR S61 OR S62 OR S63 OR S64 OR S65 OR S66 OR S67 OR S68 | 6,441,506 |
| S68 | TI "body mass index" OR AB "body mass index" | 212,706 |
| S67 | TI "neck circumference" OR AB "neck circumference" | 1,582 |
| S66 | TI "waist circumference" OR AB "waist circumference" | 31,149 |
| S65 | (MH "Body Mass Index") OR (MH "Overweight+") | 333,781 |
| S64 | TI BMI OR AB BMI | 171,212 |
| S63 | TI overweight OR AB overweight | 79,388 |
| S62 | TI adiposity OR AB adiposity | 27,626 |
| S61 | TI "body weight" OR AB "body weight" | 221,166 |
| S60 | (MH "Obesity+") OR (MH "Pediatric Obesity") | 242,859 |
| S59 | TI Obesity OR AB Obesity | 275,070 |
| S58 | (MH "Epidemiology+") | 28,059 |
| S57 | TI epidemiolog* OR AB epidemiolog* | 416,895 |
| S56 | TI occur* OR AB occur* | 2,321,486 |
| S55 | TI "odds ratio" OR AB "odds ratio" | 266,873 |
| S54 | (MH "Incidence") | 291,746 |
| S53 | TI incidence OR AB incidence | 857,530 |
| S52 | (MH "Prevalence") OR (MH "Cross-Sectional Studies") | 688,221 |
| S51 | TI prevalence OR AB prevalence | 723,389 |
| S50 | TI risk OR AB risk | 2,591,611 |

| | | |
|---|---|---|
| S49 | S20 OR S21 OR S22 OR S23 OR S24 OR S25 OR S26 OR S27 OR S28 OR S29 OR S30 OR S31 OR S32 OR S33 OR S34 OR S35 OR S36 OR S37 OR S38 OR S39 OR S40 OR S41 OR S42 OR S43 OR S44 OR S45 OR S46 OR S47 OR S48 | 4,085,319 |
| S48 | TI "musculoskeletal problems" OR AB "musculoskeletal problems" | 1,395 |
| S47 | (MH "Dyslipidemias+") | 84,673 |
| S46 | TI dyslipidaemia OR AB dyslipidaemia | 6,248 |
| S45 | TI "respiratory problem" OR AB "respiratory problem" | 200 |
| S44 | TI asthma OR AB asthma | 157,905 |
| S43 | TI anxiety OR AB anxiety | 230,285 |
| S42 | TI "psychological problem" OR AB "psychological problem" | 386 |
| S41 | TI NAFLD OR AB NAFLD | 19,045 |
| S40 | TI "non-alcoholic fatty liver disease" OR AB "non-alcoholic fatty liver disease" | 12,110 |
| S39 | TI "metabolic syndrome" OR AB "metabolic syndrome" | 56,690 |
| S38 | TI comorbid* OR AB comorbid* | 196,285 |
| S37 | TI "COMORBID*N5CHILDHOOD OBESITY" OR AB "COMORBID*N5CHILDHOOD OBESITY" | 0 |
| S36 | TI "Sleep disorders" OR AB "Sleep disorders" | 12,973 |
| S35 | TI "Sleep apnea" OR AB "Sleep apnea" | 34,927 |
| S34 | (MM "Depression") OR (MM "Depressive Disorder+") | 168,421 |
| S33 | TI Depression OR AB Depression | 381,663 |
| S32 | TI "self esteem" OR AB "self esteem" | 22,928 |
| S31 | TI "cardiac diseas*" OR AB "cardiac diseas*" | 21,256 |
| S30 | TI "heart disease*" OR AB "heart disease*" | 178,901 |
| S29 | TI CVD OR AB CVD | 44,892 |
| S28 | (MH "Cardiovascular Diseases+") | 2,609,334 |
| S27 | TI "cardiovascular disease" OR AB "cardiovascular disease" | 145,501 |
| S26 | TI "high blood pressure" OR AB "high blood pressure" | 16,270 |
| S25 | (MH "Hypertension+") | 306,040 |
| S24 | TI hypertension OR AB hypertension | 417,175 |

| | | |
|---|---|---|
| S23 | TI ( "typ* 2 diabetes mellitus" or "typ* II diabetes mellitus" ) OR AB ( "typ* 2 diabetes mellitus" or "typ* II diabetes mellitus" ) | 54,032 |
| S22 | TI "non insulin* depend* diabetes mellitus" OR AB "non insulin* depend* diabetes mellitus" | 6,887 |
| S21 | (MH "Diabetes Mellitus+") OR (MH "Diabetes Mellitus, Type 2+") | 476,881 |
| S20 | TI ( MODY or NIDDM or T2DM or T2D ) OR AB ( MODY or NIDDM or T2DM or T2D ) | 48,350 |
| S19 | S1 OR S2 OR S3 OR S4 OR S5 OR S6 OR S7 OR S8 OR S9 OR S10 OR S11 OR S12 OR S13 OR S14 OR S15 OR S16 OR S17 OR S18 | 4,222,888 |
| S18 | TI teen OR AB teen | 11,136 |
| S17 | TI preteen OR AB preteen | 311 |
| S16 | TI juvenile OR AB juvenile | 88,368 |
| S15 | TI "school age*" OR AB "school age*" | 25,638 |
| S14 | TI girls OR AB girls | 160,626 |
| S13 | TI boy* OR AB boy* | 165,930 |
| S12 | TI youth OR AB youth | 84,499 |
| S11 | (MH "Pupil") | 10,098 |
| S10 | TI "school pupil*" OR AB "school pupil*" | 1,186 |
| S9 | (MH "Students+") | 153,376 |
| S8 | TI students OR students | 406,604 |
| S7 | TI paediatric OR AB paediatric | 68,830 |
| S6 | (MH "Pediatrics+") | 62,293 |
| S5 | TI pediatric OR AB pediatric | 323,631 |
| S4 | (MH "Adolescent") | 2,170,837 |
| S3 | TI adolescen* OR AB adolescen* | 316,011 |
| S2 | (MH "Child+") | 2,070,387 |
| S1 | TI child* OR AB child* | |
| | | |
| **Table 2: Searches in CINAHL database** | | |
| # | Query | Results |

| | | |
|---|---|---|
| S71 | S19 AND S49 AND S69 AND S70 | 1,065 |
| S70 | S50 OR S51 OR S52 OR S53 OR S54 OR S55 OR S56 OR S57 OR S58 | 1,770,671 |
| S69 | S50 OR S51 OR S52 OR S53 OR S54 OR S55 OR S56 OR S57 OR S58 OR S59 OR S60 OR S61 OR S62 OR S63 OR S64 OR S65 OR S66 OR S67 OR S68 | 1,907,154 |
| S68 | TI "body mass index" OR AB "body mass index" | 75,109 |
| S67 | TI "neck circumference" OR AB "neck circumference" | 505 |
| S66 | TI "waist circumference" OR AB "waist circumference" | 11,981 |
| S65 | (MH "Body Mass Index") OR (MH "Overweight+") | 91,505 |
| S64 | TI BMI OR AB BMI | 61,729 |
| S63 | TI overweight OR AB overweight | 36,290 |
| S62 | TI adiposity OR AB adiposity | 9,276 |
| S61 | TI "body weight" OR AB "body weight" | 33,536 |
| S60 | (MH "Obesity+") OR (MH "Pediatric Obesity") | 111,008 |
| S59 | TI Obesity OR AB Obesity | 93,430 |
| S58 | (MH "Epidemiology+") | 786,283 |
| S57 | TI epidemiolog* OR AB epidemiolog* | 90,765 |
| S56 | TI occur* OR AB occur* | 329,676 |
| S55 | TI "odds ratio" OR AB "odds ratio" | 96,337 |
| S54 | (MH "Incidence") | 78,841 |
| S53 | TI incidence OR AB incidence | 193,274 |
| S52 | (MH "Prevalence") OR (MH "Cross-Sectional Studies") | 104,689 |
| S51 | TI prevalence OR AB prevalence | 215,288 |
| S50 | TI risk OR AB risk | 847,336 |
| S49 | S20 OR S21 OR S22 OR S23 OR S24 OR S25 OR S26 OR S27 OR S28 OR S29 OR S30 OR S31 OR S32 OR S33 OR S34 OR S35 OR S36 OR S37 OR S38 OR S39 OR S40 OR S41 OR S42 OR S43 OR S44 OR S45 OR S46 OR S47 OR S48 | 1,152,870 |
| S48 | TI "musculoskeletal problems" OR AB "musculoskeletal problems" | 769 |
| S47 | (MH "Dyslipidemias+") | 0 |

| | | |
|---|---|---|
| S46 | TI dyslipidaemia OR AB dyslipidaemia | 1,562 |
| S45 | TI "respiratory problem" OR AB "respiratory problem" | 52 |
| S44 | TI asthma OR AB asthma | 39,978 |
| S43 | TI anxiety OR AB anxiety | 95,902 |
| S42 | TI "psychological problem" OR AB "psychological problem" | 180 |
| S41 | TI NAFLD OR AB NAFLD | 3,821 |
| S40 | TI "non-alcoholic fatty liver disease" OR AB "non-alcoholic fatty liver disease" | 2,107 |
| S39 | TI "metabolic syndrome" OR AB "metabolic syndrome" | 16,855 |
| S38 | TI comorbid* OR AB comorbid* | 72,048 |
| S37 | TI "COMORBID*N5CHILDHOOD OBESITY" OR AB "COMORBID*N5CHILDHOOD OBESITY" | 0 |
| S36 | TI "Sleep disorders" OR AB "Sleep disorders" | 4,272 |
| S35 | TI "Sleep apnea" OR AB "Sleep apnea" | 12,516 |
| S34 | (MM "Depression") OR (MM "Depressive Disorder+") | 72,746 |
| S33 | TI Depression OR AB Depression | 141,871 |
| S32 | TI "self esteem" OR AB "self esteem" | 13,914 |
| S31 | TI "cardiac diseas*" OR AB "cardiac diseas*" | 4,598 |
| S30 | TI "heart disease*" OR AB "heart disease*" | 47,503 |
| S29 | TI CVD OR AB CVD | 14,948 |
| S28 | (MH "Cardiovascular Diseases+") | 656,720 |
| S27 | TI "cardiovascular disease" OR AB "cardiovascular disease" | 49,301 |
| S26 | TI "high blood pressure" OR AB "high blood pressure" | 5,654 |
| S25 | (MH "Hypertension+") | 89,092 |
| S24 | TI hypertension OR AB hypertension | 98,430 |
| S23 | TI ( "typ* 2 diabetes mellitus" or "typ* II diabetes mellitus" ) OR AB ( "typ* 2 diabetes mellitus" or "typ* II diabetes mellitus" ) | 16,533 |
| S22 | TI "non insulin* depend* diabetes mellitus" OR AB "non insulin* depend* diabetes mellitus" | 916 |
| S21 | (MH "Diabetes Mellitus+") OR (MH "Diabetes Mellitus, Type 2+") | 184,018 |

| | | |
|---|---|---|
| S20 | TI ( MODY or NIDDM or T2DM or T2D ) OR AB ( MODY or NIDDM or T2DM or T2D ) | 13,615 |
| S19 | S1 OR S2 OR S3 OR S4 OR S5 OR S6 OR S7 OR S8 OR S9 OR S10 OR S11 OR S12 OR S13 OR S14 OR S15 OR S16 OR S17 OR S18 | 1,296,760 |
| S18 | TI teen OR AB teen | 10,026 |
| S17 | TI preteen OR AB preteen | 222 |
| S16 | TI juvenile OR AB juvenile | 11,490 |
| S15 | TI "school age*" OR AB "school age*" | 12,324 |
| S14 | TI girls OR AB girls | 48,902 |
| S13 | TI boy* OR AB boy* | 47,998 |
| S12 | TI youth OR AB youth | 57,833 |
| S11 | (MH "Pupil") | 1,132 |
| S10 | TI "school pupil*" OR AB "school pupil*" | 439 |
| S9 | (MH "Students+") | 152,969 |
| S8 | TI students OR students | 264,459 |
| S7 | TI paediatric OR AB paediatric | 154,344 |
| S6 | (MH "Pediatrics+") | 22,940 |
| S5 | TI pediatric OR AB pediatric | 154,344 |
| S4 | (MH "Adolescent") | 0 |
| S3 | TI adolescen* OR AB adolescen* | 155,786 |
| S2 | (MH "Child+") | 733,676 |
| S1 | TI child* OR AB child* | 557,797 |

**Table 3: Searches in Embase database**

| # | Query | Results |
|---|---|---|
| 1 | child/ or juvenile/ or boy/ or girl/ or school child/ | 1666492 |
| 2 | exp adolescent/ | 1331618 |
| 3 | Pediatric*.mp. | 584553 |
| 4 | Paediatric.mp. or exp pediatrics/ | 198524 |
| 5 | exp student/ | 270289 |

| | | |
|---|---|---|
| 6 | student/ or exp controlled study/ or School pupils.mp. or exp school/ | 8733161 |
| 7 | Youth.mp. or juvenile/ | 113110 |
| 8 | Boys.mp. or boy/ | 122142 |
| 9 | Girls.mp. or girl/ | 134388 |
| 10 | Preteen.mp. | 219 |
| 11 | Teen.mp. | 7208 |
| 12 | 1 or 2 or 3 or 4 or 5 or 6 or 7 or 8 or 9 or 10 or 11 | 10570321 |
| 13 | Obesity.mp. or obesity/ | 592346 |
| 14 | body weight.mp. or body weight/ | 512252 |
| 15 | adiposity.mp. or obesity/ | 462778 |
| 16 | body mass/ or body weight/ or over weight.mp. | 770604 |
| 17 | BMI.mp. | 360243 |
| 18 | body mass index.mp. or body mass/ | 576219 |
| 19 | waist circumference.mp. or waist circumference/ | 70311 |
| 20 | Neck circumference.mp. or neck circumference/ | 4192 |
| 21 | 13 or 14 or 15 or 16 or 17 or 18 or 19 or 20 | 1391889 |
| 22 | Diabetes Mellitus.mp. or diabetes mellitus/ | 958620 |
| 23 | (TMODY or NIDDM or T2DM or T2D).mp. | 76297 |
| 24 | (non insulin* depend*or noninsulin* depend* or non insulindepend* or noninsulindepend*).mp. | 71 |
| 25 | adolescent depression/ or Depression.mp. or depression/ | 671230 |
| 26 | Self esteem.mp. or self esteem/ | 33542 |
| 27 | Sleep apnea.mp. or sleep disordered breathing/ | 90388 |
| 28 | Sleep disorders.mp. or sleep disorder/ | 80158 |
| 29 | hypertension.mp. or hypertension/ | 847577 |
| 30 | high blood pressure.mp. or hypertension/ | 573026 |
| 31 | Cardiovascular Diseases.mp. or cardiovascular disease/ | 338213 |
| 32 | CVD.mp. | 68314 |
| 33 | heart disease.mp. or heart disease/ | 353094 |

| | | |
|---|---|---|
| 34 | cardiac disease.mp. or heart disease/ | 115398 |
| 35 | comorbidity/ or Comorbid*.mp. | 491271 |
| 36 | psychological problem.mp. | 507 |
| 37 | anxiety/ or anxiety.mp. | 396144 |
| 38 | exp asthma/ or asthma.mp. | 265979 |
| 39 | respiratory tract disease/ or respiratory problem.mp. | 59094 |
| 40 | dyslipidaemia.mp. or dyslipidemia/ | 89074 |
| 41 | musculoskeletal disease/ or musculoskeletal problems.mp. | 31740 |
| 42 | 22 or 23 or 24 or 25 or 26 or 27 or 28 or 29 or 30 or 31 or 32 or 33 or 34 or 35 or 36 or 37 or 38 or 39 or 40 or 41 | 3482359 |
| 43 | prevalence/ or Prevalence.mp. | 1164303 |
| 44 | incidence/ or incidence.mp. | 1208070 |
| 45 | odds ratio.mp. or odds ratio/ or risk/ | 770430 |
| 46 | risk ratio.mp. | 29795 |
| 47 | occurrence.mp. | 444269 |
| 48 | epidemiology/ or epidemiology.mp. | 1221041 |
| 49 | 43 or 44 or 45 or 46 or 47 or 48 | 3802476 |
| 50 | 12 and 21 and 42 and 49 | 1207 |

**Table 4: Searches in PsycInfo database**

| # | Query | Results |
|---|---|---|
| S61 | S17 AND S28 AND S53 AND S60 | 351 |
| S60 | S54 OR S55 OR S56 OR S57 OR S58 OR S59 | 261,550 |
| S59 | TI epidemiology OR AB epidemiology | 16,841 |
| S58 | TI occurrence OR AB occurrence | 52,005 |
| S57 | TI risk ratio OR AB risk ratio | 6,584 |
| S56 | TI odds ratio OR AB odds ratio | 33,534 |
| S55 | TI incidence OR AB incidence | 54,925 |
| S54 | TI Prevalence OR AB Prevalence | 128,349 |
| S53 | S29 OR S30 OR S31 OR S32 OR S33 OR S34 OR S35 OR S36 OR S37 OR S38 OR S39 OR S40 OR S41 OR S42 OR S43 OR | 574,649 |

| | | |
|---|---|---|
| | S44 OR S45 OR S46 OR S47 OR S48 OR S49 OR S50 OR S51 OR S52 | |
| S52 | TI musculoskeletal problems OR AB musculoskeletal problems | 389 |
| S51 | TI dyslipidaemia OR AB dyslipidaemia | 215 |
| S50 | TI respiratory problem OR AB respiratory problem | 467 |
| S49 | DE "Asthma" | 5,643 |
| S48 | TI asthma OR AB asthma | 7,758 |
| S47 | TI sleep apnoea OR AB sleep apnoea | 519 |
| S46 | DE "Self-Esteem" OR DE "Self-Confidence" | 30,982 |
| S45 | DE "Anxiety" OR DE "Anxiety Disorders" | 115,286 |
| S44 | TI self-esteem OR AB self-esteem | 46,484 |
| S43 | TI anxiety OR AB anxiety | 217,475 |
| S42 | DE "Behavior Problems" | 31,222 |
| S41 | TI psychological problem OR AB psychological problem | 15,447 |
| S40 | DE "Depression (Emotion)" | 26,562 |
| S39 | TI depression OR AB depression | 267,913 |
| S38 | TI NAFLD OR AB NAFLD | 126 |
| S37 | TI non-alcoholic fatty liver disease OR AB non-alcoholic fatty liver disease | 81 |
| S36 | TI metabolic syndrome OR AB metabolic syndrome | 3,850 |
| S35 | TI CVD OR AB CVD | 3,297 |
| S34 | TI cardiovascular disease OR AB cardiovascular disease | 13,300 |
| S33 | TI high blood pressure OR AB high blood pressure | 17,760 |
| S32 | TI hypertension OR AB hypertension | 17,760 |
| S31 | TI Type 2 Diabetes Mellitus OR AB Type 2 Diabetes Mellitus | 2,314 |
| S30 | DE "Comorbidity" OR DE "Sequelae" | 60,083 |
| S29 | TI Comorbidity OR AB Comordidity | 6,509 |
| S28 | S18 OR S19 OR S20 OR S21 OR S22 OR S23 OR S24 OR S25 OR S26 OR S27 | 80,444 |
| S27 | TI BMI OR AB BMI | 20,049 |
| S26 | TI neck circumference OR AB neck circumference | 114 |

| | | |
|---|---|---|
| S25 | TI waist circumference OR AB waist circumference | 3,135 |
| S24 | DE "Body Mass Index" | 17,962 |
| S23 | TI body mass index OR AB body mass index | 22,249 |
| S22 | DE "Body Fat" OR DE "Adipocytes" | 2,474 |
| S21 | TI adiposity OR AB adiposity | 2,377 |
| S20 | TI body weight OR AB body weight | 21,115 |
| S19 | DE "Obesity" OR DE "Body Mass Index" | 42,762 |
| S18 | TI obesity OR AB obesity | 35,659 |
| S17 | S1 OR S2 OR S3 OR S4 OR S5 OR S6 OR S7 OR S8 OR S9 OR S10 OR S11 OR S12 OR S13 OR S14 OR S15 OR S16 | 1,772,269 |
| S16 | TI (preteens or youth or adolescents or teen or teenagers) OR AB (preteens or youth or adolescents or teen or teenagers) | 310,451 |
| S15 | TI juvenile OR AB juvenile | 26,731 |
| S14 | TI school age OR AB school age | 24,229 |
| S13 | DE "Human Females" | 94,610 |
| S12 | TI girls OR AB girls | 77,239 |
| S11 | DE "Human Males" | 28,255 |
| S10 | TI boys OR AB boys | 76,287 |
| S9 | TI youth OR AB youth | 109,629 |
| S8 | TI "school pupils" OR AB "school pupils" | 2,236 |
| S7 | TI youth OR AB youth | 109,629 |
| S6 | TI youth OR AB youth | 109,629 |
| S5 | DE "Students" OR DE "Primary School Students" OR DE "Junior High School Students" | 68,448 |
| S4 | TI students OR students | 716,207 |
| S3 | TI paediatric OR paediatric | 105,188 |
| S2 | TI child* OR AB child* | 742,326 |
| S1 | TX adolescents OR AB adolescents | 505,004 |
| **Table 5: Searches in Scopus database** | | |
| # | Query | Results |

| | | |
|---|---|---|
| 1 | ((children OR adolescents OR paediatric OR students OR school pupils OR youth OR boys OR girls OR school age OR juvenile OR preteens OR teens) AND (obesity OR body AND weight OR adiposity OR “body mass index” OR “waist circumference” OR “neck circumference”) AND (comorbidity OR “type 2 diabetes mellitus” OR hypertension OR “high blood pressure” OR “cardiovascular disease” OR cvd OR “metabolic syndrome” OR “non-alcoholic fatty liver disease” OR NAFLD OR depression OR psychological problem” OR anxiety OR self-esteem OR “sleep apnoea” OR asthma OR respiratory problem” OR dyslipidaemia OR “musculoskeletal problems”) AND (prevalence OR incidence OR “odds ratio” OR “risk ratio” OR occurrence OR epidemiology)) | 205 |

**Table 6: Searches in Web of Science database**

| # | Query | Results |
|---|---|---|
| 1 | (children or adolescents or paediatric or students or school pupils or youth or boys or girls or school age or juvenile or preteens or teens) AND (obesity or body weight or adiposity or body mass index or waist circumference or neck circumference) AND (Comorbidity or Type 2 Diabetes Mellitus or hypertension or high blood pressure or cardiovascular disease or CVD or metabolic syndrome or non-alcoholic fatty liver disease or NAFLD or depression or psychological problem or anxiety or self-esteem or sleep apnoea or asthma or respiratory problem or dyslipidaemia or musculoskeletal problems) AND (Prevalence or incidence or odds ratio or risk ratio or occurrence or epidemiology) | 117 |
